# Supplementary figures and images for: Identifying factors associated with vaping cessation in young adults: A machine learning and XAI approach
Source: PLOS Digit Health. 2026 May 5;5(5):e0001031. doi: 10.1371/journal.pdig.0001031 (PMC13143109; doi:10.1371/journal.pdig.0001031)

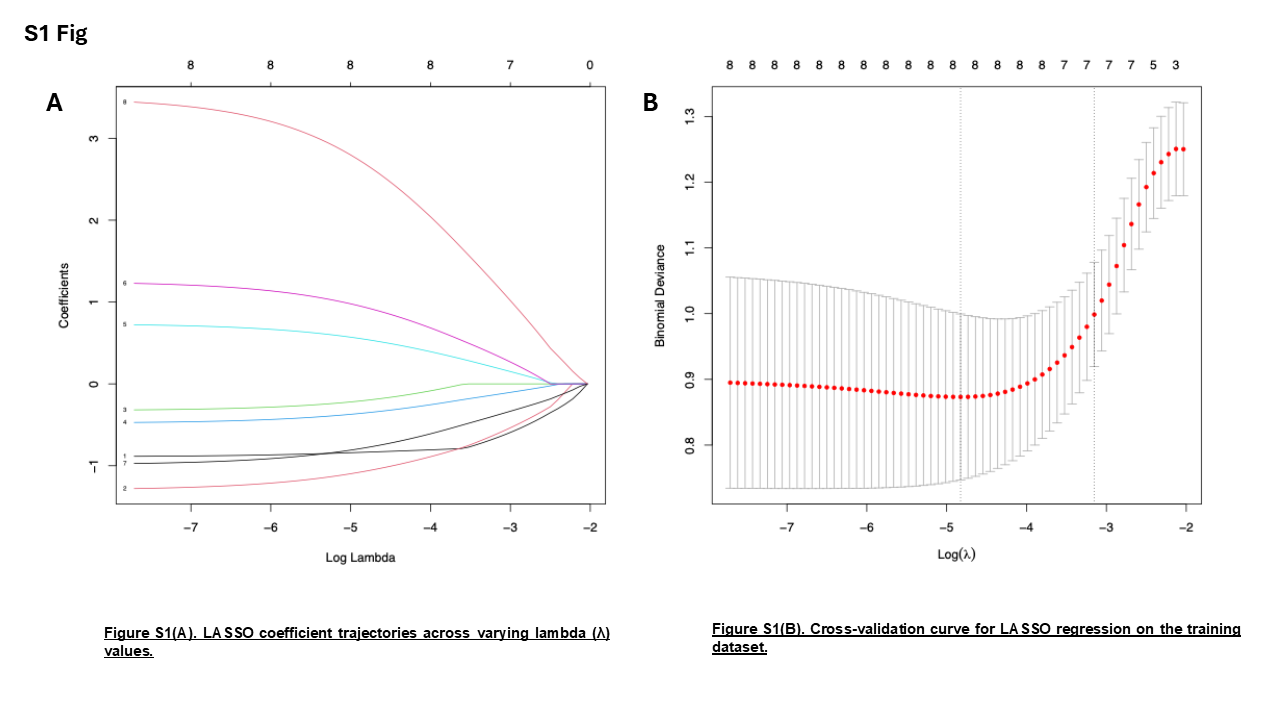

Supplement: S1 Fig — This plot illustrates how the estimated coefficients of predictors change as the regularization parameter λ increases. As λ grows, the model imposes stronger penalization, driving more coefficients to zero and resulting in increasingly sparse models that retain only the most influential predictors. (B). Cross-validation curve for LASSO regression on the training dataset. The plot shows the 10-fold cross-validated binomial deviance as a function of log-transformed lambda (λ), the regularization tuning parameter. This visualization supports model optimization by identifying the λ value that minimizes deviance, thereby selecting the most effective level of sparsity for the LASSO-regularized model. (TIF) [file pdig.0001031.s001.tif]

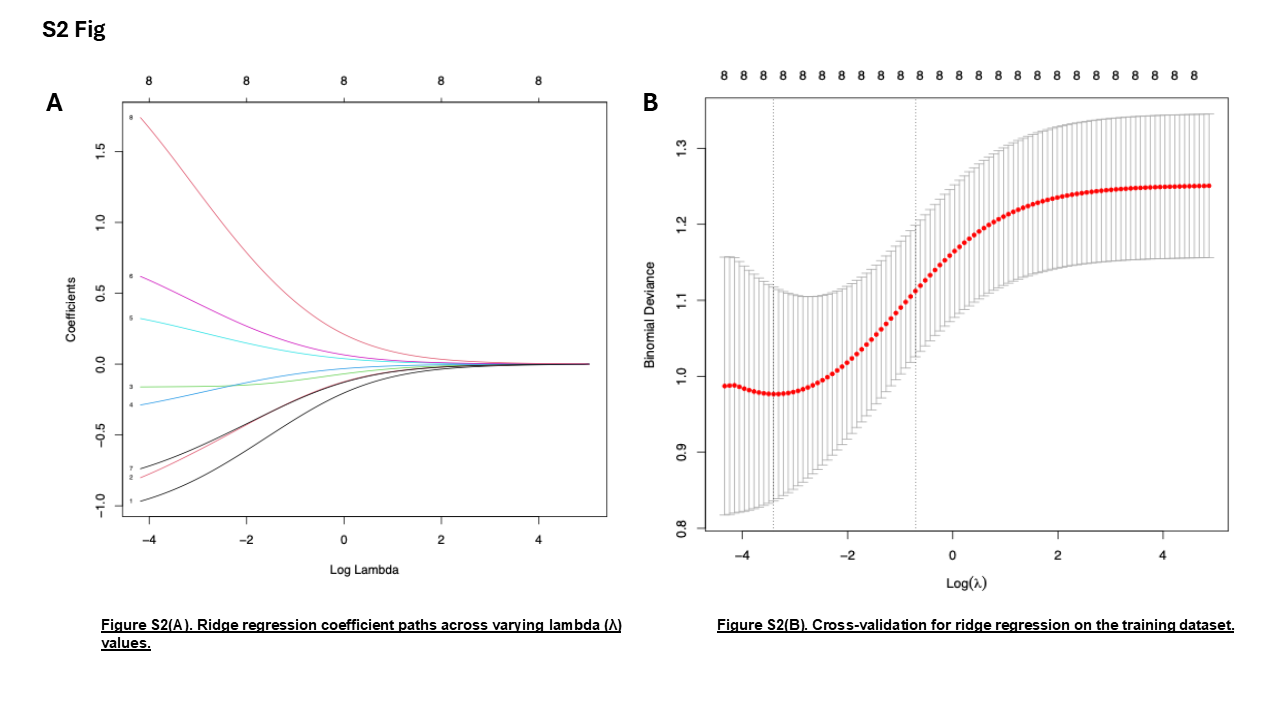

Supplement: S2 Fig — This plot illustrates how the estimated coefficients of predictors evolve as the regularization parameter λ increases. As λ grows, the model increasingly penalizes complexity, causing the coefficients to shrink toward zero—resulting in progressively sparser models with fewer influential predictors. (B) Cross-validation for ridge regression on the training dataset. The plot displays the 10-fold cross-validated binomial deviance as a function of log-transformed lambda (λ), the regularization tuning parameter. Although labeled for ridge regression, the curve reflects performance metrics for a lasso-regularized model, aiding in the selection of the optimal λ that minimizes deviance and enhances model generalization. (TIF) [file pdig.0001031.s002.tif]

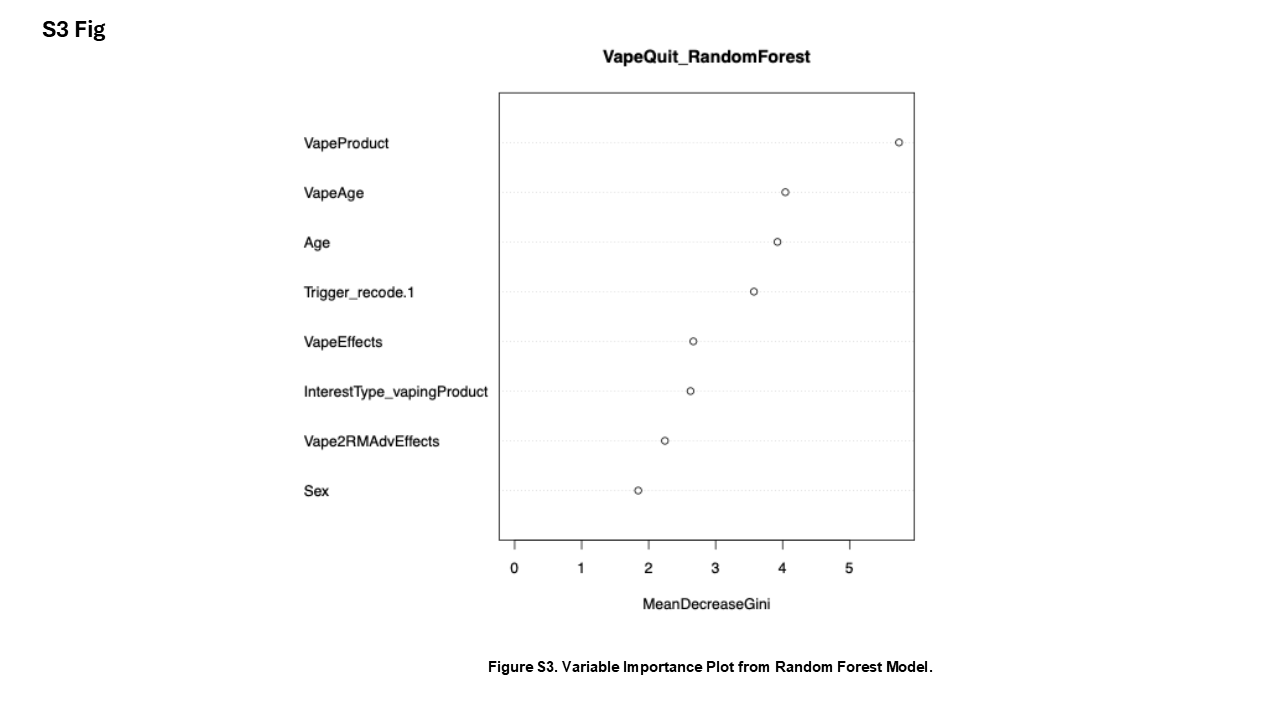

Supplement: S3 Fig — The plot displays the relative importance of predictor variables based on Mean Decrease Accuracy and Mean Decrease Gini. Variables are ranked from most to least important, with higher values indicating greater contribution to model performance. (TIF) [file pdig.0001031.s003.tif]
