# Supplementary material for: Identifying factors associated with vaping cessation in young adults: A machine learning and XAI approach
Source: PLOS Digit Health. 2026 May 5;5(5):e0001031. doi: 10.1371/journal.pdig.0001031 (PMC13143109; doi:10.1371/journal.pdig.0001031)
Supplement: Attachment — Submitted filename: 1.docx [file pdig.0001031.s004.docx]

- Very small sample size (n = 119) for complex ML and XAI models; this is insufficient for algorithms such as Random Forest, SVM, and Elastic Net, making the results highly prone to overfitting.
- Non-random, social-media–based convenience sampling, which introduces severe selection bias and limits the generalizability of the findings.
- Insufficient information about the questionnaire (no details about validation, reliability, use of standardized scales, etc.).
- Outcome variable ("vaping cessation") is poorly defined—no information on how quitting was measured (self-report? duration of abstinence?).
- Mismatch between study purpose and design: the authors aim to “predict” outcomes, yet the study is cross-sectional; true prediction requires longitudinal data.
- Important confounders not controlled in modeling.
- No external validation dataset, relying only on a small train/test split from the same sample.
- Unrealistic and inconsistent model performance results (e.g., Random Forest AUC = 0.99 in training but 0.70 in testing), clearly indicating extreme overfitting, but the manuscript does not address this.
- Incorrect or unclear reporting of model parameters:
  - “Lambda” mentioned for Random Forest, which is conceptually incorrect.
  - RF outputs are treated as if they were regression coefficients, which is wrong.
- Inconsistencies between methods and results: Boruta, LIME, and ALE are mentioned, but explanations and figures are incomplete or poorly integrated.
- XAI component is vague and lacks transparency; implementation details, exact parameters, and interpretability logic are not clearly described.
- Key ML performance metrics are missing, including:

Precision, Recall

F1-score

Confusion matrix

Calibration curves

- No handling of class imbalance, and the proportion of cessation vs. non-cessation cases is not reported.
- Excessive descriptive statistics with long paragraphs and limited relevance to the predictive aim.
- No clear tables summarizing the main model outputs (only AUC and Brier Score).
- Interpretations are speculative and sometimes contradictory, without empirical justification.
- No confidence intervals (CIs) reported for AUC, Brier Score, or model coefficients.
- Overly long, general, and repetitive discussion, resembling a narrative review rather than a focused interpretation of findings.
- Overstated claims about the effectiveness and policy implications of ML despite weak data.
- Conclusions exceed what the data can support, given the small sample and methodological flaws.
- Lack of acknowledgment of major limitations, such as:

Self-report bias

Cross-sectional design

Convenience sampling

Overfitting

Limited generalizability

- Long paragraphs, redundant explanations, and inconsistent writing style.
- Conceptual errors in explaining statistical methods, such as treating RF parameters like linear coefficients.
- Introduction is overly lengthy and distracts from the research question.
- Manuscript does not fully follow PLOS Digital Health’s structural expectations, particularly for ML reporting.
- Inconsistencies between figure legends and the actual figures.
- Some figures lack adequate explanation or contain incorrect interpretations.
- Mismatch between described variables and what appears in the figures (e.g., Figure 4 mentions 26 predictors but shows only 6).
