# Supplementary material for: Identifying factors associated with vaping cessation in young adults: A machine learning and XAI approach
Source: PLOS Digit Health. 2026 May 5;5(5):e0001031. doi: 10.1371/journal.pdig.0001031 (PMC13143109; doi:10.1371/journal.pdig.0001031)
Supplement: Attachment — Submitted filename: reviews.docx [file pdig.0001031.s005.docx]

**Review Comments to the Author**

This manuscript explores the use of machine learning and explainable AI to predict vaping cessation among young adults, addressing a highly relevant public health challenge. The topic is well aligned with PLOS Digital Health, and the authors demonstrate familiarity with a broad range of ML techniques and contemporary cessation literature. Ethical approval and participant protections are appropriately described.

That said, several major issues should be addressed before the manuscript can be considered for publication:

The abstract should be rewritten in a structured format consistent with PLOS Digital Health standards. Specifically, the authors should clearly separate and label the following components: Background, Objective, Methods, Results, and Conclusions. The current abstract is narrative and overly dense, making it difficult to quickly identify the study aim, analytic approach, and key findings. A structured abstract would substantially improve clarity, accessibility, and alignment with journal expectations, particularly for a digitally focused and methodologically complex study.

Language: The manuscript is generally intelligible and written in standard academic English. However, substantial editorial refinement is needed: Repetition across the Introduction and Discussion reduces clarity and conciseness. Some sections (especially Results and Discussion) are overly long and descriptive, with limited synthesis. Terminology is occasionally inconsistent (e.g., cessation definitions, model performance claims).
Minor grammatical errors, formatting inconsistencies, and typographical issues are present.

Outcome Definition and Study Design: The primary outcome of “vaping cessation” is insufficiently defined. It is unclear whether cessation reflects a quit attempt, short-term abstinence, or sustained cessation, and over what timeframe. This ambiguity undermines interpretation of model performance and clinical relevance.

Sample Size and Model Complexity
The relatively small sample (n = 119) is a major limitation given the number of predictors and the use of complex models (Random Forest, SVM, XAI). The manuscript should explicitly discuss risks of overfitting, limited generalizability, and instability of feature importance rankings.

Statistical and ML Methodology: While multiple models are explored, the rationale for using overlapping feature-selection strategies (forward/backward selection, Boruta, LASSO) is unclear. Performance comparisons between models are sometimes inconsistent, and claims of superiority should be tempered. Calibration results indicating borderline or poor fit require discussion.

Explainability and XAI Claims: The manuscript emphasizes explainable AI, yet the practical contribution of ALE and LIME analyses to public health decision-making is not clearly articulated. The authors should clarify how these explanations add value beyond traditional regression outputs.

Data Availability and Reproducibility: The current Data Availability Statement does not meet PLOS requirements. Public deposition of de-identified data and analytic code is essential for transparency and reproducibility.

Interpretation and Scope of Conclusions: Several conclusions particularly regarding sex differences, behavioral readiness, and intervention design extend beyond what can be supported by this dataset. These interpretations should be reframed as exploratory and hypothesis-generating.

Manuscript Structure and Clarity: The manuscript would benefit from substantial condensation, especially in the Discussion, and clearer separation between results, interpretation, and implications.

This is a promising and relevant study with potential contribution to digital public health research. However, substantial revisions are required to strengthen methodological rigor, transparency, and alignment between data, analyses, and conclusions.
