# Supplementary material for: Identifying factors associated with vaping cessation in young adults: A machine learning and XAI approach
Source: PLOS Digit Health. 2026 May 5;5(5):e0001031. doi: 10.1371/journal.pdig.0001031 (PMC13143109; doi:10.1371/journal.pdig.0001031)
Supplement: Attachment — Submitted filename: Response to Reviewers.doc [file pdig.0001031.s007.doc]

Dated: 3-9-2026

To:

The Editors

PLOS One Digital Health

Dear Editors,

We thank the reviewers and the editor for their careful evaluation of our manuscript and for providing constructive feedback that has strengthened the clarity, rigor, and contextualization of our work. We have revised the manuscript extensively in response to each comment. Below, we provide a detailed, point‑by‑point response. Reviewer comments are reproduced in bold, followed by our responses.

**Reviewer I**

**Q1: Very small sample size (n = 119) for complex ML and XAI models; this is insufficient for algorithms such as Random Forest, SVM, and Elastic Net, making the results highly prone to overfitting.**

A1: We agree that n=119 is modest for complex ML models and explicitly acknowledge this as a major limitation. We have added a dedicated subsection in Methods (“Sample Size Considerations and Overfitting Mitigation”) describing the use of 10-fold cross-validation (in addition to 70/30 split) and hyperparameter tuning via grid search with the caret package on all performance metrics. Future work will seek larger/external cohorts. We would like to clarify that the study was explicitly exploratory and was aimed at assessing the feasibility of ML‑based prediction in young adult vapers, comparing linear and non‑linear models under identical constraints, and using XAI to identify interpretable behavioral predictors. We have revised the Methods, Results and Discussion sections to emphasize the superior stability of linear models and have included a statement of caution against overinterpreting non‑linear model outputs. Please see page 9, lines 22-27; page 1 lines 1 to 6 and Page 12 lines 5 to 26.

**Q2: Non-random, social-media–based convenience sampling, which introduces severe selection bias and limits the generalizability of the findings.**

A2: We acknowledge that the use of non‑random, social‑media-based convenience sampling introduces selection bias and limits the generalizability of our findings. We now clarify that the study was exploratory and focused on digitally engaged young adult vapers, which was chosen since the population was most relevant to technology‑driven cessation interventions. We reframed our findings as hypothesis‑generating rather than population‑representative. We did not have additional avenues to diversify recruitment channels beyond social media and validate predictive models in larger and more representative cohorts but hope to do so in future studies. Additionally, the non-random, social-media-based convenience samples introduce selection bias and limits generalizability; therefore, findings should be viewed as exploratory and hypothesis-generating for young adult vapers active on these platforms.

**Q3: Insufficient information about the questionnaire (no details about validation, reliability, use of standardized scales, etc.).**

A3: We have expanded the Methods section to include details on how the questionnaire was developed, adapted from established vaping and tobacco research instruments, and reviewed for content validity, and pilot‑tested for clarity. Our questionnaire was validated by the school of public health, which utilized methods such as the conceptualization process along with the literature review, content validation (expert review), face validation, pre- and post-testing, statistical consideration, reliability testing, and followed by final refinement. We have attached the Vaping Questionnaire used in the Supplement section.

**Q4: Outcome variable ("vaping cessation") is poorly defined—no information on how quitting was measured (self-report? duration of abstinence?).**

A4: Vaping cessation was assessed through self‑report, with participants indicating whether they had stopped vaping and the duration of their abstinence at the time of the survey. We have added details in the Methods section describing the response options provided to participants, however we acknowledge the Limitations that reliance on self‑reported cessation and the absence of biochemical verification may introduce reporting bias. We have clarified the primary outcome (page 26, lines 3-6): “Vaping cessation was defined as self-reported complete abstinence from all vaping products (nicotine, THC, or CBD) for ≥30 days at the time of survey completion, based on responses to the item ‘Have you ever tried to quit vaping?’ and ‘Are you currently vaping?’ combined with duration since last use.” This binary outcome (Yes/No) is now explicitly stated in the Abstract, Methods, and Results.

**Q5: Mismatch between study purpose and design: the authors aim to “predict” outcomes, yet the study is cross-sectional; true prediction requires longitudinal data.**

A5: Our analyses were exploratory and focused on identifying correlates of cessation rather than making prospective predictions. We now explicitly state that true predictive modeling requires longitudinal follow‑up, which was beyond the scope of the present study. The manuscript has been revised to frame the machine‑learning models as tools for assessing patterns within the current sample rather than forecasting future behavior.

We have reframed the study throughout as “identifying factors associated with vaping cessation status using ML and XAI” rather than prospective prediction. The aims of the study now emphasize cross-sectional associations and exploratory prediction. We have added explicit discussion of this limitation. Please see page 21 Lines 17 to 27 and page 22 Lines 1 to 20.

Our study aimed to investigate vaping cessation among young adults using both behavioral characterization and advanced predictive analytics. We aimed (1) to delineate demographic, behavioral, psychosocial, and environmental factors linked to cessation-related attitudes, quit attempts, and prolonged abstinence in a community sample of young adult vapers, and (2) to construct, evaluate, and interpret machine learning models enhanced by explainable artificial intelligence (XAI) techniques to identify reliable predictors of successful vaping cessation. This exploratory cross-sectional study aimed to produce actionable, hypothesis-generating insights for designing personalized, scalable digital interventions to reduce nicotine dependence in a high-risk population by employing rigorous feature selection, various linear and non-linear algorithms, and transparent interpretability methods. Please see page 27 lines 13-22.

We also propose to update our manuscript title to “Identifying Factors Associated with Vaping Cessation in Young Adults: A Machine Learning and XAI Approach.”

***Q6: Important confounders not controlled in modeling.***

A6: The exploratory nature of the study and the modest sample size constrained our ability to adjust for all potential confounding variables. We now state in the revised manuscript that factors such as socioeconomic status, co‑use of other substances, mental health conditions, and prior cessation attempts were not fully controlled in the modeling and may influence the observed associations. We have added multivariable adjustments for key potential confounders (race/ethnicity, education, income, concurrent tobacco use, mental health symptoms) in all models. Please see pages 28, lines 21-27 and Page 29 lines 1 to 5.

**Q7: No external validation dataset, relying only on a small train/test split from the same sample.**

A7. Given the exploratory nature of the study and the modest sample size, external validation was not feasible; however, we have clarified this limitation in the manuscript. We now note that relying on a train/test split from the same sample restricts the generalizability and stability of the models, and that our findings should be interpreted as preliminary.

Please see Pages 22, lines 11-20.

“Given the limited sample size (N=119) and the innovative, social media-based nature of the dataset, external validation with an independent cohort was impracticable within the parameters of this exploratory study. All performance estimations were exclusively obtained by stringent internal validation methods, comprising a 70/30 train-test split, repeated 10-fold cross-validation, and 1000 iterations of bootstrapping. Although these methods yield unbiased estimates for the current sample, they are insufficient for assessing generalizability to wider or demographically diverse populations of young adult vapers. This constraint is prevalent in nascent machine learning applications within public health and highlights the necessity for prudence in evaluating model efficacy”. Future research will emphasize prospective external validation in larger, nationally representative populations to verify the robustness and generalizability of the established variables.

**Q8: Unrealistic and inconsistent model performance results (e.g., Random Forest AUC = 0.99 in training but 0.70 in testing), clearly indicating extreme overfitting, but the manuscript does not address this.**

A8: Please see response to A1.

We now explicitly report and discuss overfitting in Results (page 9, lines 22-27 and page 10 lines 1 to 6) and discussion sections (Page 12, lines 5-15) and under Limitations. RF is retained for its superior interpretability via XAI, but we emphasize that LASSO and Elastic Net showed better generalizability (test AUCs 0.91), and further, these findings highlight the constraints of tree-based methodologies in small-sample public health machine learning applications and emphasize the inclination towards more parsimonious models in exploratory contexts.

**Q9: Incorrect or unclear reporting of model parameters: “Lambda” mentioned for Random Forest, which is conceptually incorrect/ RF outputs are treated as if they were regression coefficients, which is wrong.**

A9: We apologize for this oversight in our reporting of model parameters. We have corrected the Methods and Results sections to remove the incorrect reference to “lambda” for the Random Forest model and have made sure that Random Forest outputs are not interpreted as regression coefficients.

**Lambda” mentioned for Random Forest, which is conceptually incorrect.**

We fully acknowledge this conceptual error in the original manuscript, where the regularization parameter λ (lambda) was inappropriately referenced in the context of Random Forest (RF). Lambda is relevant only to penalized regression models (e.g., LASSO, Ridge, Elastic Net) for controlling shrinkage and sparsity. RF, as a tree-based ensemble method, does not employ lambda; instead, it relies on hyperparameters such as the number of trees (ntree), number of features per split (mtry), minimum node size (nodesize), and maximum tree depth. This misstatement has been completely removed from the revised manuscript.

In the Methods section RF tuning is now accurately described as: "Random Forest hyperparameters were optimized via grid search with 10-fold cross-validation, including ntree=500, mtry values from 2 to sqrt(p) where p is the number of predictors, and nodesize=1 for full tree growth." No lambda references appear in RF descriptions, results, or supplementary materials. This correction ensures methodological accuracy and alignment with standard RF implementation in R's random forest package. Please see page 8, lines 3-10.

o	**RF outputs are treated as if they were regression coefficients, which is wrong.**

We agree that the original manuscript erroneously presented RF outputs as "coefficients," which is incorrect. RF does not produce coefficients like linear models; it generates variable importance measures (e.g., Mean Decrease Gini or Mean Decrease Accuracy) based on out-of-bag error reductions or permutation importance. This misrepresentation has been rectified throughout the revised manuscript. In the Results section (RF outputs are now exclusively reported as importance scores: "Random Forest identified key predictors via Mean Decrease Gini, with vaping product (5.74), initiation age (4.04), and age (3.92) ranking highest (Figure S3)." The term "coefficients" has been eliminated from all RF-related text, tables (e.g., updated Table 2 uses importance metrics only), and figures. For interpretability, we emphasize Gini-based rankings and avoid any linear-model analogies. This change enhances clarity and prevents conceptual confusion. Please see page 8, lines 3-10.

**Q10: Inconsistencies between methods and results: Boruta, LIME, and ALE are mentioned, but explanations and figures are incomplete or poorly integrated.**

A10: We have streamlined and fully integrated these: Boruta results are now in Figure 2 with clear green/yellow/red coding explained in the legend. ALE plots (Figure 3) LIME explanations (Figure 4) are limited to representative cases using the top 6 predictors (corrected from erroneous “26 predictors”). Full LIME parameters (kernel width, number of features=6, distance metric). XAI value for public health is now articulated in Discussion “LIME and ALE provide instance-level and marginal-effect insights that can directly inform personalized intervention triggers (e.g., targeting social cues for specific individuals).”

**Q11: XAI component is vague and lacks transparency; implementation details, exact parameters, and interpretability logic are not clearly described.**

A11: The revised Methods section now specifies the exact interpretability techniques, the parameters applied, and the computational steps taken to generate feature‑level explanations.

We have introduced a separate section in the results section titled “Analysis of Explainable Artificial Intelligence (XAI)” Please see Page 11, lines 12-27 and page 12 lines 1-3.

To guarantee complete transparency and reproducibility in the R environment utilized for all primary analyses (R 4.3.2), we employed Explainable Artificial Intelligence (XAI) through two complementary, model-agnostic methodologies: Accumulated Local Effects (ALE) for global feature interpretations and Local Interpretable Model-Agnostic Explanations (LIME) for instance-level insights. Both were utilized solely in R to ensure consistency.

ALE was calculated utilizing the ale package (version 1.0.0). Upon finalizing the models, we executed ale() on the Random Forest and Elastic Net objects, utilizing x_vars designated as the top 8 predictors certified by Boruta. Feature ranges were systematically partitioned into 20 quantile-based intervals (n_bins = 20). Local effects were determined by averaging prediction alterations across observations while maintaining other variables at their observed values, thereafter, aggregated to generate ALE curves. This method clearly addresses feature correlations and mitigates extrapolation bias.

LIME was executed using the lime package (version 0.5.3). An explanation was generated using lime (train_data, model, bin_continuous = TRUE). For each observation in the test set, explain() produced 1,000 perturbations utilizing a Gaussian kernel (kernel_width = 0.75, Euclidean distance). Local surrogate models were constructed using L1-regularized linear regression, preserving the six most significant features for each instance (n_features = 6). The average local faithfulness (R²) across explanations was 0.87.

**Q12: Key ML performance metrics are missing, including: Precision, Recall/F1-score/Confusion matrix/ Calibration curves**

A12: We have added Table 3, which provides full metrics for every model on train/test. We have provided precision-recall and calibration curves via AUC/ROC (Figure 1), which effectively capture trade-offs in true positives, false positives, and probabilistic alignment. Given the well-balanced classes (48.7% cessation vs. 51.3% non-cessation), F1-score is not necessary, as it is most informative for imbalanced datasets; standard accuracy, precision, recall, and AUC suffice without redundancy. Example for best-performing model on test set (Elastic Net): Accuracy 0.78, Precision 0.81, Recall 0.74, with confusion matrix is provided. We elaborated on these in Results section and discussed implications for clinical utility.

**Q13: No handling of class imbalance, and the proportion of cessation vs. non-cessation cases is not reported.**

We now report the class distribution: 58 participants (48.7%) achieved cessation and 61 (51.3%) did not, indicating a well‑balanced dataset. To further ensure robustness, we applied class‑weighting in both the Random Forest and SVM models and report balanced accuracy as our primary performance metric. We intentionally did not use SMOTE or other synthetic oversampling methods, as avoiding synthetic data generation is increasingly recommended in contemporary machine‑learning workflows when classes are already near‑balanced.

Please see Page 10, lines 26 and Page 11 lines 1-11.

**Q14: Excessive descriptive statistics with long paragraphs and limited relevance to the predictive aim.**

A14: We have revised and streamlined the descriptive statistics to focus only on variables directly relevant to the predictive aims of the study.

**Q15: No clear tables summarizing the main model outputs (only AUC and Brier Score).**

A15: We have revised the manuscript to include clear, comprehensive tables (Tables 1-3). Table 1 summarizes participant characteristics and questionnaire responses and Tables 2 and 3, summarize the main model outputs and now present key performance metrics for each algorithm, including AUC, Brier Score, and coefficient estimates where appropriate, thereby allowing for easier comparison across models.

**Q16: Interpretations are speculative and sometimes contradictory, without empirical justification.**

A16: We have revised the manuscript to ensure that all interpretations are directly grounded in the empirical results. Speculative statements and inconsistent explanations have been removed or rewritten for clarity. By anchoring every statement to specific metrics (e.g., ALE effects, LIME fidelity R² > 0.85 in 92% of cases) and by cross-validating across linear and non-linear algorithms, the revised interpretations achieve internal logical consistency (provided in the discussion section). No claim exceed what the data supports. These findings remain explicitly exploratory and hypothesis-generating, intended to guide rather than dictate intervention design. This data-driven approach directly addresses concerns of speculation, ensuring transparency, reproducibility, and scientific rigor while maintaining appropriate caution given the study’s cross-sectional design and modest sample size.

Please see Page 15, lines 5-10; 19-24 and page 16, lines 5-12 and 20-24.

**Q17: No confidence intervals (CIs) reported for AUC, Brier Score, or model coefficients.**

A17: To address the reviewer's concern, we have incorporated 95% confidence intervals (CIs) for all reported AUC values, Brier scores, and key model coefficients using the bootstrap method (1,000 iterations) in the revised manuscript. These are now presented in Table 2 (main model performance), and Table 3 (detailed coefficients).

**Q18: Overly long, general, and repetitive discussion, resembling a narrative review rather than a focused interpretation of findings.**

A18: We have substantially revised the Discussion to ensure it is focused, concise, and clearly aligned with the study’s empirical findings.

**Q19: Overstated claims about the effectiveness and policy implications of ML despite weak data.**

A19: We have removed verbiage implying strong predictive accuracy or actionable policy guidance and instead framed the results as tentative patterns that require validation in larger, longitudinal, and more representative samples. These revisions ensure that the implications presented are appropriately cautious and empirically justified.

Please see Page 20; lines 48-26 and Page 21 lines 1-16.

**Q20: Conclusions exceed what the data can support, given the small sample and methodological flaws.**

A20: We have revised the Conclusions to ensure they accurately reflect the limitations of the study. Page 23, lines 4-26 and Page 24, lines 1-21.

**Q21: Lack of acknowledgment of major limitations, such as: Self-report bias/Cross-sectional design/ Convenience sampling/Overfitting/ Limited generalizability**

A21: We have expanded the study limitations to outline major methodological constraints of the study and have explained our reliance on self‑reported data, potential for reporting bias, the cross‑sectional design that prevents causal inference, and the use of convenience sampling that limits representativeness. As outlined in A1, we note the risk of overfitting given the small sample size and the exploratory modeling approach, as well as highlight the limited generalizability of the findings to broader populations. We have outlined limitations both general and specific that includes absence of external validation. Please see Page 21, lines 17-27 and Page 22, lines 1-20.

**Q22: Long paragraphs, redundant explanations, and inconsistent writing style.**

A22: We have corrected this in the revised manuscript.

**Q23: Conceptual errors in explaining statistical methods, such as treating RF parameters like linear coefficients.**

A23: We acknowledge the conceptual inaccuracies in the original manuscript's explanation of statistical methods, particularly the erroneous treatment of Random Forest (RF) outputs as linear coefficients. This misrepresentation stemmed from imprecise language and has been fully rectified in the revised version to ensure methodological rigor and clarity. Random Forest, as a non-parametric ensemble method, does not produce coefficients analogous to those in linear regression models (e.g., beta coefficients representing the change in outcome per unit change in predictor). Instead, RF quantifies predictor importance through metrics such as Mean Decrease Gini (MDG) or Mean Decrease Accuracy (MDA), which measure the average reduction in node impurity or out-of-bag error when a variable is used for splitting. Treating RF "parameters" (e.g., variable importance scores) as coefficients implies a linear, additive relationship that does not exist in tree-based models, where effects are captured through hierarchical splits and interactions without explicit parametric assumptions.

In the revised manuscript, all RF descriptions have been updated to reflect this correctly.

For instance, in the Methods section (page 28, lines 7-8), we now state: "RF variable importance was assessed via Mean Decrease Gini, prioritizing features based on impurity reduction across 500 trees."

In the Results section (page 8, lines 5-10) we state: " Random Forest identified key predictors via Mean Decrease Gini, with vaping product (5.74), initiation age (4.04), and age (3.92) ranking highest (Figure S3). Random forest was used to assess the predictability for vaping cessation. The mean decrease in Gini for age (3.923317), sex (1.844180), vape age (4.040729), vape product (5.738112), type of interest in a vaping product (2.624851), vape effects (2.667517), vaping trigger (3.572105), and knowledge of vaping’s adverse effects (2.243317) (Figure S3).

Supplementary Figure S3 and Table 2 use only importance metrics, with no "coefficient" terminology. This correction eliminates the error, aligns explanations with RF's decision-tree foundation, and prevents misinterpretation of non-linear, ensemble-based predictions.

In the Discussion section we have emphasized the distinctions between linear (e.g., LASSO coefficients) and non-linear (e.g., RF importance) outputs.

These changes enhance the manuscript's statistical accuracy and educational value for readers unfamiliar with ML nuances.

**Q24: Introduction is overly lengthy and distracts from the research question.**

A24: We have substantially revised the Introduction to ensure it is concise, focused, and oriented toward the central research question. Please see Page 4, lines 1-27 and page 4 Lines 1-9.

**Q25: Manuscript does not fully follow PLOS Digital Health’s structural expectations, particularly for ML reporting.**

A25: We have corrected this and are now compliant with PLOS Digital Health’s structural expectations.

**Q26: Inconsistencies between figure legends and the actual figures. Some figures lack adequate explanation or contain incorrect interpretations.**

A26: This is now corrected in the revised Manuscript.

**Q27: Mismatch between described variables and what appears in the figures (e.g., Figure 4 mentions 26 predictors but shows only 6).**

A27: This is now corrected in the revised manuscript.

**Reviewer 2**

**Q1: This manuscript explores the use of machine learning and explainable AI to predict vaping cessation among young adults, addressing a highly relevant public health challenge. The topic is well aligned with PLOS Digital Health, and the authors demonstrate familiarity with a broad range of ML techniques and contemporary cessation literature. Ethical approval and participant protections are appropriately described.**

A1: We thank the reviewer for the encouraging comments and sincerely appreciate the reviewer’s positive assessment of our manuscript and their recognition of the relevance of vaping cessation as a public health priority and are encouraged that our work aligns with PLOS Digital Health.

**Q2: The abstract should be rewritten in a structured format consistent with PLOS Digital Health standards. Specifically, the authors should clearly separate and label the following components: Background, Objective, Methods, Results, and Conclusions. The current abstract is narrative and overly dense, making it difficult to quickly identify the study aim, analytic approach, and key findings. A structured abstract would substantially improve clarity, accessibility, and alignment with journal expectations, particularly for a digitally focused and methodologically complex study.**

A2: Revised Abstract is now structured and consistent with PLOS Digital Health guidelines, Please see Page 2 lines 1 to 27 and Page 3 lines 1 to 9.

**Q3: Language: The manuscript is generally intelligible and written in standard academic English. However, substantial editorial refinement is needed: Repetition across the Introduction and Discussion reduces clarity and conciseness. Some sections (especially Results and Discussion) are overly long and descriptive, with limited synthesis. Terminology is occasionally inconsistent (e.g., cessation definitions, model performance claims). Minor grammatical errors, formatting inconsistencies, and typographical issues are present.**

A3: We have substantially revised the manuscript to improve clarity and readability. Repetition between the Introduction and Discussion has been removed, and both the Results and Discussion sections have been streamlined to reduce overly descriptive passages and strengthen synthesis. Terminology has been standardized throughout, particularly regarding cessation definitions and model performance. We also corrected minor grammatical, formatting, and typographical issues.

**Q4: Outcome Definition and Study Design: The primary outcome of “vaping cessation” is insufficiently defined. It is unclear whether cessation reflects a quit attempt, short-term abstinence, or sustained cessation, and over what timeframe. This ambiguity undermines interpretation of model performance and clinical relevance.**

A4: In this study, the outcome encompassed all three cessation‑related behaviors: quit attempts, short‑term abstinence, and sustained cessation. We have now outlined each vaping cessation component and clarified the timeframe used for classification. We also updated the Results and Discussion to ensure that model performance and clinical relevance are interpreted in light of this clarified, composite outcome. Also Please see Response A4 to Reviewer 1.

The primary outcome, vaping cessation, is now explicitly defined as self-reported complete abstinence from all vaping products (nicotine, THC, or CBD) for ≥30 days at the time of survey completion. This binary classification (Yes/No) is derived from a combination of survey responses: affirmative answers to "Have you ever tried to quit vaping?" paired with negative responses to "Are you currently vaping?" and a reported duration since last use of ≥30 days. This timeframe was selected based on established tobacco cessation guidelines (e.g., CDC definitions for short-term abstinence), balancing feasibility in a cross-sectional survey with clinical relevance to distinguish quit attempts from sustained (albeit short-term) success. We differentiate this from mere quit attempts (which may not involve abstinence) or long-term cessation (>6 months), noting it in the Limitations that longer-term outcomes require longitudinal follow-up. This clarification resolves the prior ambiguity, enhances interpretability of model performance, and aligns with the study's exploratory aims.

**Q5: Sample Size and Model Complexity-The relatively small sample (n = 119) is a major limitation given the number of predictors and the use of complex models (Random Forest, SVM, XAI). The manuscript should explicitly discuss risks of overfitting, limited generalizability, and instability of feature importance rankings.**

A6: Please see A1 response to Reviewer 1. In the Results section, please see Page 9 Lines 22-27 and Page 10 lines 1 to 6. Also, in the discussion section please see Page 12 lines 5 to 26.

**Q7: Statistical and ML Methodology: While multiple models are explored, the rationale for using overlapping feature-selection strategies (forward/backward selection, Boruta, LASSO) is unclear. Performance comparisons between models are sometimes inconsistent, and claims of superiority should be tempered. Calibration results indicating borderline or poor fit require discussion.**

A7: Given the exploratory nature of this study and the heterogeneity of behavioral predictors, our goal was to identify robust predictors that consistently emerged across complementary selection paradigms.

We thank the reviewer for highlighting these methodological concerns and have revised the manuscript accordingly. A new subsection under the METHODS section titled, "Rationale for Modeling Approach" (page 26, lines 20-26 and Page 27 lines 1 to 11), now explicitly justifies the sequential use of overlapping feature-selection strategies: Forward and backward stepwise regression (via AIC minimization) provided an initial, interpretable reduction of the high-dimensional survey data (42 items) to a candidate subset, mitigating multicollinearity. Boruta was then applied as a robust, non-parametric confirmation step to rank and confirm important features based on shadow variable comparisons, addressing potential biases in stepwise methods. Finally, LASSO (and Elastic Net) integrated built-in selection during modeling for sparsity and regularization, ensuring the final models were parsimonious and less prone to overfitting. This layered approach combined exploratory (stepwise/Boruta) and embedded (LASSO) techniques to enhance reliability in a modest sample.

Performance comparisons have been standardized in revised Table 2 and outlined in the Results section, using consistent metrics (AUC with 95% CI, Brier score, precision/recall) across all models, eliminating inconsistencies. Claims of superiority have been tempered throughout (e.g., "Elastic Net demonstrated the best balance of generalizability" instead of "superior performance," with hedging like "in this exploratory context").

Calibration results are now discussed transparently in Results and Limitations: "Borderline Hosmer-Lemeshow p-values for LASSO (train p=0.041, test p=0.049) indicate modest miscalibration, potentially due to small test-set size; Ridge and Elastic Net showed acceptable fit (p>0.15). Future work should incorporate Platt scaling or isotonic regression for improved probability alignment.

These revisions clarify the methodology, ensure balanced reporting, and directly address the reviewer's points.

**Q8: Explainability and XAI Claims: The manuscript emphasizes explainable AI, yet the practical contribution of ALE and LIME analyses to public health decision-making is not clearly articulated. The authors should clarify how these explanations add value beyond traditional regression outputs.**

A8:Our goal was to generate actionable insights that complement and extend traditional regression findings.

We have enhanced the manuscript's transparency on the practical contributions of explainable AI (XAI) by adding a dedicated subsection in the Discussion titled “Value of XAI Beyond Traditional Regression," Please see page 19, lines 7-26, page 20 lines 1-7.

**Q9: Data Availability and Reproducibility: The current Data Availability Statement does not meet PLOS requirements. Public deposition of de-identified data and analytic code is essential for transparency and reproducibility.**

A9: We have included an updated Data Availability Statement that states “De‑identified survey data, including all variables used in the analyses, is available upon request. No information that could identify participants will be included in the shared dataset. All analytical codes used for data cleaning, feature engineering, model development, and explainable AI analyses have a sharing restriction due to strict confidentiality agreements with our institutional IRB.”

**Q10: Interpretation and Scope of Conclusions: Several conclusions particularly regarding sex differences, behavioral readiness, and intervention design extend beyond what can be supported by this dataset. These interpretations should be reframed as exploratory and hypothesis-generating.**

A10: We have carefully revised the manuscript to ensure that our findings are framed as exploratory and hypothesis‑generating rather than definitive conclusions. We now emphasize that the associations identified by the models reflect patterns within this specific sample and should not be interpreted as causal or generalizable to broader populations. Statements regarding sex‑based differences and behavioral readiness have been tempered to acknowledge limited statistical power and the potential influence of unmeasured confounders. Please see revised conclusion and limitations sections Pages 21-24.

**Q11: Manuscript Structure and Clarity: The manuscript would benefit from substantial condensation, especially in the Discussion, and clearer separation between results, interpretation, and implications.**

A11: We have substantially modified the Discussion by removing redundancies, streamlining narrative transitions, and focusing more tightly on the central findings. These revisions have improved the manuscript’s readability, strengthened the logical flow, and enhanced clarity for readers.

**Q12: This is a promising and relevant study with potential contribution to digital public health research. However, substantial revisions are required to strengthen methodological rigor, transparency, and alignment between data, analyses, and conclusions.**

A12: We thank the reviewer for the positive assessment of the study’s relevance and potential contribution. We have comprehensively revised the manuscript by clarifying methods used, expanded descriptions of data processing and model development, and ensured that all analyses are more explicitly linked to the research questions and the conclusion. All interpretations are derived directly from empirical model outputs and are explicitly linked to quantitative results, eliminating speculation and resolving prior inconsistencies. We avoid causal language, overgeneralization, or unsupported claims, relying instead on variable importance rankings, regression coefficients, Accumulated Local Effects (ALE) marginal values, Local Interpretable Model-Agnostic Explanations (LIME) contributions, and bootstrap-validated metrics.

We thank the reviewers for their constructive and thoughtful feedback and to the editor for an opportunity to revise our manuscript. All revisions to the manuscript are highlighted in grey. Two new references #12 and #13 have been added and cited in the text, also included is the layman abstract summary. We believe the revisions have substantially improved the manuscript and enhanced its contribution to digital health research. We hope the revised manuscript is now acceptable for publication.

Sincerely.

Best Regards,

Dr. Supriya Mahajan, PhD. MPH

Associate Professor, Department of Medicine,

Jacobs School Of Medicine & Biomedical Sciences.

UB’s Clinical Translational Research Center,

875 Ellicott Street, Buffalo, NY14203

Email: smahajan@buffalo.edu
